# MGST1 alleviates the oxidative stress of trophoblast cells induced by hypoxia/reoxygenation and promotes cell proliferation, migration, and invasion by activating the PI3K/AKT/mTOR pathway

**DOI:** 10.1515/med-2022-0617

**Published:** 2022-12-14

**Authors:** Hu Dai, Xianmei Lu

**Affiliations:** Department of Obstetrics and Gynecology, Hongsheng Community Health Service Center, Wuxi, Jiangsu, 214111, China; Department of Medical Center of Diagnosis and Treatment for Cervical Diseases, The Affiliated Wuxi Maternity and Child Health Care Hospital of Nanjing Medical University, No. 48, Huaishu Lane, Liangxi District, Wuxi, Jiangsu, 214000, China

**Keywords:** preeclampsia, MGST1, PI3K/AKT/mTOR pathway, oxidative stress

## Abstract

Preeclampsia (PE) is a common pregnancy-specific syndrome with an incidence of 4.6% in all pregnant women. Numerous studies have uncovered the functions and mechanisms of microsomal glutathione transferase 1 (MGST1) in different diseases and cellular processes, but whether MGST1 plays a role in PE remains unclear. Our study aimed to investigate the regulatory role of MGST1 in PE progression. In this study, the HTR8/SVneo cells were incubated with CoCl_2_ (250 µM) to mimic hypoxia in trophoblasts. Real-time quantitative polymerase chain reaction revealed that MGST1 was dramatically reduced in the placenta of PE patients. The proliferation of HTR8/SVneo cells was assessed via the Cell Counting Kit-8 and colony formation assays, and the results showed that MGST1 upregulation increased the cell viability of HTR8/SVneo cells. In addition, wound healing and Transwell assays unveiled that the elevation of MGST1 enhanced trophoblast cell migration and invasion. Moreover, the upregulation of MGST1 alleviated the hypoxia-induced oxidative stress in trophoblast cell. Mechanically, we found that MGST1 regulated PE progression by activating the phosphoinositide-3-kinase/protein kinase B/mechanistic target of rapamycin (PI3K/AKT/mTOR) pathway. In conclusion, MGST1 alleviated the oxidative stress of trophoblast cells induced by hypoxia/reoxygenation and promoted cell proliferation, migration, and invasion via the activation of the PI3K/AKT/mTOR pathway in PE. These results suggested that MGST1 can be a potential target for the prevention and treatment of PE.

## 1 Introduction

Preeclampsia (PE) is a common pregnancy-specific syndrome with an incidence of 4.6% in all pregnant women [1]. PE usually occurs during the second or third trimester of gestation and is characterized by hypertension and proteinuria [2,3]. PE has become the leading cause of preterm delivery and maternal mortality all over the world [4,5]. It has been reported that normal deep placentation is highly associated with the differentiation and invasion ability of trophoblast cells during gestation [6]. Dysregulation of trophoblast cell activities may be a cause of PE, and it has been confirmed that excessive trophoblast cell apoptosis was closely correlated with PE development, which was considered to be an important pathogenesis of PE [7]. Therefore, more and more studies focus on the trophoblast cells in PE.

Microsomal glutathione transferase 1 (MGST1) is a protein-coding gene with 39,102 nucleotides and is located at 12p12.3 [8]. MGST1 is a membrane-related protein in eicosanoid and glutathione (GSH) metabolism [9]. There were six types of proteins in membrane-related proteins in eicosanoid and GSH metabolism, and glutathione S-transferases (GSTs) are one of them [10]. As a subtype of GSTs, MGST1 is reported to be implicated in cell defense against carcinogenic, toxic, and pharmacologically active electrophilic compounds [11]. Previously, a review from Schaffert indicated that MGST1 is involved in reactive, intermediate-induced injury [12]. MGST1 is also identified as playing a protective role in the retinal pigment epithelium against oxidative stress and aging [13]. MGST1 participates in the growth, migration, invasion, and epithelial–mesenchymal transition of glioma cells by acting as a target of miR-379-5p [14]. Another study also demonstrated that MGST1 silencing attenuated the proliferation and promoted the apoptosis of lung adenocarcinoma cells [15]. Despite the numerous findings about the functions and mechanisms of MGST1 in different diseases and cellular processes, whether MGST1 serves a part in PE remains largely unclear.

This study aimed to probe the regulatory role of MGST1 in PE progression. Our work is the first time to reveal that MGST1 alleviated the oxidative stress of trophoblast cells induced by hypoxia/reoxygenation and accelerated cell proliferation, migration, and invasion through the activation of phosphoinositide-3-kinase/protein kinase B/mechanistic target of rapamycin (PI3K/AKT/mTOR) pathway in PE. The findings might shed some light on the role of MGST1 in the prevention and treatment of PE.

## 2 Methods

### 2.1 Participants and tissue sample collection

In total, 40 pregnant women from Hongsheng Community Health Service Center were enrolled in our study (PE group [*n* = 20], normal group [*n* = 20]). PE was diagnosed in line with the criteria of the American College of Obstetrics and Gynecology. The placental tissues were immediately collected after delivery and stored at −80°C post-washing.


**Ethics approval:** Ethical approval was obtained from the Ethics Committee of the Hongsheng Community Health Service Center.
**Statement of informed consent:** Written informed consent was obtained from a legally authorized representative(s) for anonymized patient information to be published in this article.
**Data availability statement:** The datasets generated during and/or analysed during the current study are available from the corresponding author on reasonable request.

### 2.2 Cell culture and hypoxia treatment

HTR8/SVneo cells were obtained from the Shanghai Institute of Biochemistry and Cell Biology (Shanghai, China) and grown in RPMI-1640 (Gibco, California, USA) with additional 10% fetal bovine serum (Gibco) in an atmosphere of 5% CO_2_ at 37°C. The cells were incubated with CoCl_2_ (250 µM) to mimic hypoxia in trophoblasts for 48 h. MGST1 was knocked down by transfecting with short interference (si) RNA siMGST1 and overexpressed by transfection with the MGST1 vector (GenePharma, Shanghai, China).

### 2.3 Transwell assay

Cell invasion of HTR8/SVneo cells was conducted using Corning Costar Transwell chambers pre-coated with Matrigel (Sigma, St Louis, USA). The transfected HTR8/SVneo cells were grown in the upper chamber with a serum-free medium, and RPMI-1640 (Gibco; 600 μL) with 10% calf serum was added in the lower chamber. After 24 h, crystal violet (1%; Sigma) was applied for staining the cells in the lower chamber cells for 20 min in 2% ethanol, and a microscope (DM6000B, Leica) was used to count the number of stained cells.

### 2.4 Real-time quantitative polymerase chain reaction (RT-qPCR)

The Trizol reagent (Thermo Fisher Scientific, Waltham, USA) was employed for the extraction of total RNA from the placental tissues of participants. The complementary DNA was synthesized via the SuperScript reverse transcriptase kit (Vazyme, Nanjing, China), and RT-qPCR was employed for evaluating the level of MGST1 on the Applied Biosystems 7500 Fast Real-Time PCR System (Applied Biosystems, Foster City, USA) according to the 2^−ΔΔCt^ method. β-Actin served as a control. The primers were listed as follows:

- MGST1
  F: 5′−AAATGGGCCAACCTGGATGT−3′
  R: 5′−ACACTGGTTTACCTGCGTACA−3′;
- β-actin
  F: 5′−GGACATCCGCAAAGACCTGTA−3′
  R: 5′−GCTCAGGAGGAGCAATGATCT−3′.

### 2.5 Western blot analysis

The proteins were isolated from tissues or cells via radio immunoprecipitation assay lysis buffer. Then, sodium dodecyl sulfate–polyacrylamide gel electrophoresis was used to separate the proteins, followed by transferring them onto the polyvinylidene difluoride membrane (Sigma). The membranes were further incubated with the respective primary antibodies for overnight, followed by incubating with a secondary antibody against rabbit IgG (1:1,000; ab190475; Abcam, Shanghai) for 1 h at indoor temperature. An enhanced chemiluminescence system (Thermo Fisher Scientific, USA) was used to detect the protein bands, and ImageJ software (National Institutes of Health, Bethesda, USA) was applied for the quantification of proteins. The primary antibodies included anti-MGST1 (1:1,000; ab131059; Abcam), anti-AKT (10 µL; ab283852; Abcam), anti-PI3K (10 µL; ab283852; Abcam), anti-mTOR (1:10,000; ab134903; Abcam), p-AKT (1:1,000; ab38449; Abcam), p-PI3K (0.5 µg/mL, ab278545; Abcam), p-mTOR (1:1,000; ab109268; Abcam), and β-actin (1 µg/mL; ab8226; Abcam).

### 2.6 Cell Counting Kit-8 (CCK-8) assay

The viability of HTR8/SVneo cells was assessed via the CCK-8 (Dojindo, Tokyo, Japan) assay. Transfected cells were plated in 96-well plates for 24 h, and CCK-8 solution (10 μl) was supplemented into the wells every 24 h. After incubation for 2 h, the 450 nm absorbance was evaluated.

### 2.7 Wound healing assay

The migration of HTR8/SVneo cells was examined by a wound healing assay. The cells were planted in six-well plates, and a 200 μL-tip was used to scratch on the plates. The migration of the cells was photographed at 0 and 48 h, and the wound healing rate was calculated based on (width at 0 h − width of the wound at 48 h)/width at 0 h.

### 2.8 Colony formation assay

The transfected HTR8/SVneo cells were grown in six-well plates with RPMI-1640 medium for 2 weeks. The cell colonies were fixed by crystal violet (0.1%) and methanol solution (20%) for 10 min. A Nikon microscope (Tokyo, Japan) was used to count and photograph the visible colonies.

### 2.9 Immunohistochemistry (IHC) assay

The placental tissues were fixed in 10% formalin and paraffin-embedded. The tissues were cut into sections, and the antigen was retrieved by ethylene diamine tetraacetic acid (Invitrogen, Carlsbad, USA). The endogenous peroxidase activity was sealed by a 0.5% hydrogen peroxide–methanol solution. The tissues were incubated with anti-MGST1 (1:1,000; ab232469; Abcam) antibodies for 2 h followed by adding secondary antibodies (1:1,000; Santa Cruz Biotechnology, USA) into it for half an hour. Next, diaminobenzidine (Zytomed Systems, Berlin, Germany) was applied for dyeing the tissues, and then the tissues were counterstained with hematoxylin (Servicebio, Wuhan, China) after washing. The nuclear staining was performed with 4,6-diamino-2-phenyl indole. The stained tissues were observed via an Olympus fluorescence microscope (Olympus Corporation, Tokyo, Japan), and the expression of MGST1 was analyzed.

### 2.10 Enzyme-linked immunosorbent assay (ELISA)

The ELISA was used for evaluating the concentrations of oxidative stress indexes, including superoxide dismutase (SOD), GSH, malondialdehyde (MDA), and myeloperoxidase (MPO) via respective ELISA kits.

### 2.11 Statistical analysis

The Gene Expression Omnibus (GEO) datasets were downloaded from national center of biotechnology information(https://www.ncbi.nlm.nih.gov/) to probe MGST1 expression in the placenta of preterm PE patients and normal preterm pregnant women. Statistical product service solutions (version 20.0, IBM, USA) was employed for data analysis of the experimental data. The data were displayed as mean ± standard deviation, and comparisons were subjected to an independent sample *t*-test or one-way analysis of variance. *P* < 0.05 was set as statistical significance. All tests were executed at least three times.

## 3 Results

### 3.1 MGST1 was lowly expressed in the placenta of PE patients

**Figure 1:**
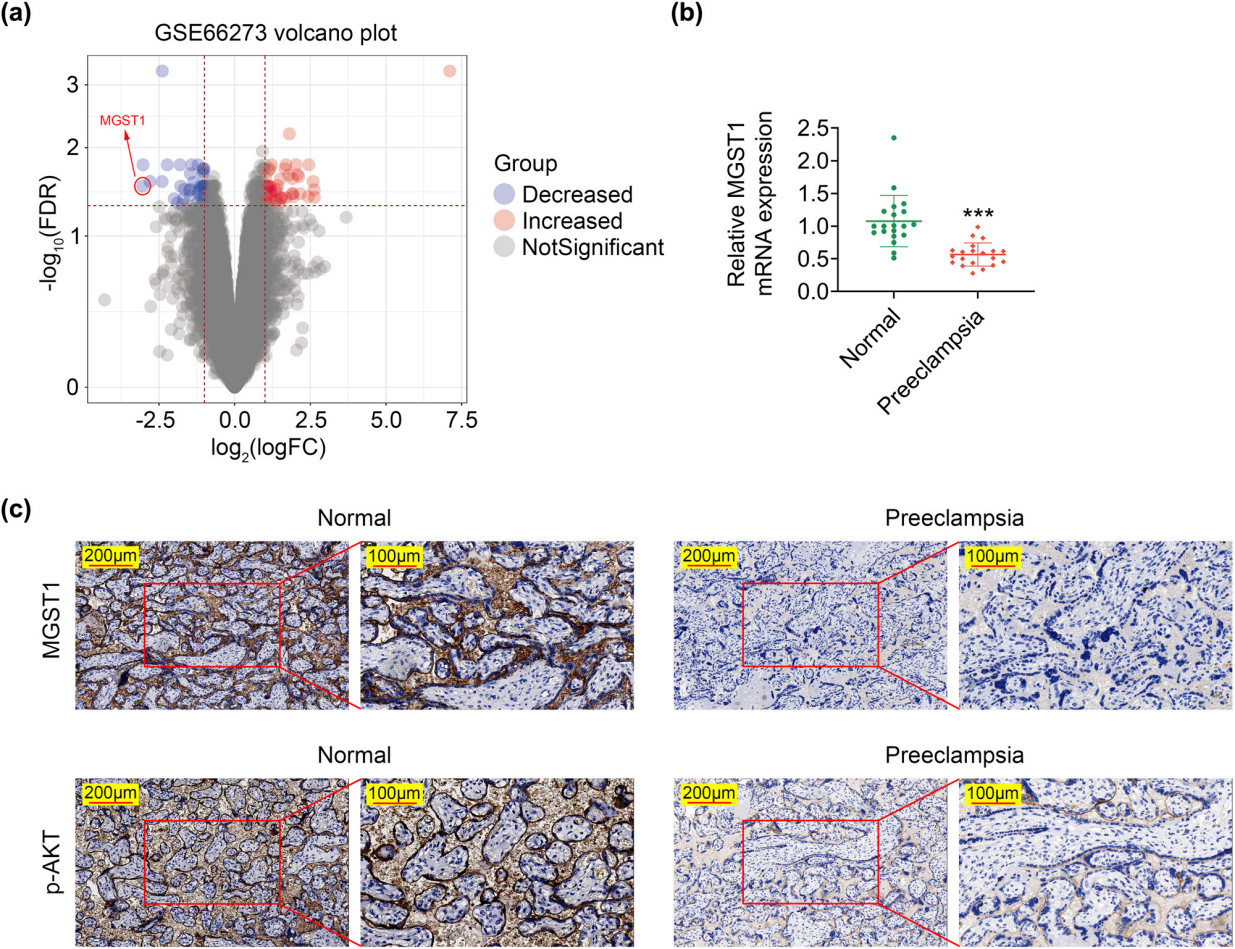
MGST1 was lowly expressed in the placenta of PE patients. (a) Volcano plot showed the expression level of MGST1 in the placenta of preterm PE and normal preterm pregnant women using GEO data. (b) RT-qPCR was applied to assess the mRNA level of MGST1. (c) IHC assay manifested the expression of MGST1 and p-AKT in the placental tissues. ^***^ *P* < 0.001.

In order to identify the role of MGST1 in PE, the level of MGST1 in the placenta of preterm PE and normal preterm pregnant women was analyzed using GEO data. The volcano plot depicted that the level of MGST1 was evidently lower in the placenta of preterm PE patients than that in normal preterm pregnant women (Figure 1a). In addition, our study recruited 20 PE patients and 20 normal pregnant women. The maternal and fetal baseline data are exhibited in Table 1. The data demonstrated that the gestational age (39.64 weeks vs 37.49 weeks), the placental weight of women (542.94 vs 512.24 g), fetal weight (3423.05 vs 3092.55 g), and length (55.97 vs 49.48 cm) in the normal group were higher than those in the PE group. The body mass index (BMI, 10.94 vs 12.65 kg/m^2^), urinary protein (0.27 g/24 h vs 2.36 g/24 h), systolic pressure (113.8 vs 145.78 mmHg), and diastolic pressure (68.89 vs 99.17 mmHg) were lower in the normal group than those in the PE group. Similarly, RT-qPCR also revealed that the MGST1 mRNA level was dramatically decreased in the placenta of PE patients (Figure 1b). IHC also showed that the MGST1 and p-AKT expressions were downregulated in the placental tissues of PE patients (Figure 1c). All in all, MGST1 was lowly expressed in the placenta of PE patients.

**Table 1:** Maternal and fetal baseline demographic data of the PE patients and normal pregnant women

Clinical indicators	Normal (*n* = 20)	PE (*n* = 20)
Maternal age (years)	30.49 ± 1.67	29.92 ± 2.15
Gestational age (weeks)	39.64 ± 0.81	37.49 ± 1.22***
BMI (kg/m^2^)	10.94 ± 1.61	12.65 ± 1.35***
Urinary protein (g/24 h)	0.27 ± 0.01	2.36 ± 0.12***
Systolic pressure (mmHg)	113.8 ± 3.36	145.78 ± 7.31***
Diastolic pressure (mmHg)	68.89 ± 8.14	99.17 ± 5.82***
Fetal weight (g)	3423.05 ± 488.29	3092.55 ± 335.92*
Fetal length (cm)	55.97 ± 1.04	49.48 ± 1.88***
Placental weight (g)	542.94 ± 44.06	512.24 ± 23.12**

*P < 0.05, **P < 0.01, ***P < 0.001.

### 3.2 Overexpression of MGST1 increased the hypoxia-induced trophoblast cell proliferation, migration, and invasion

**Figure 2:**
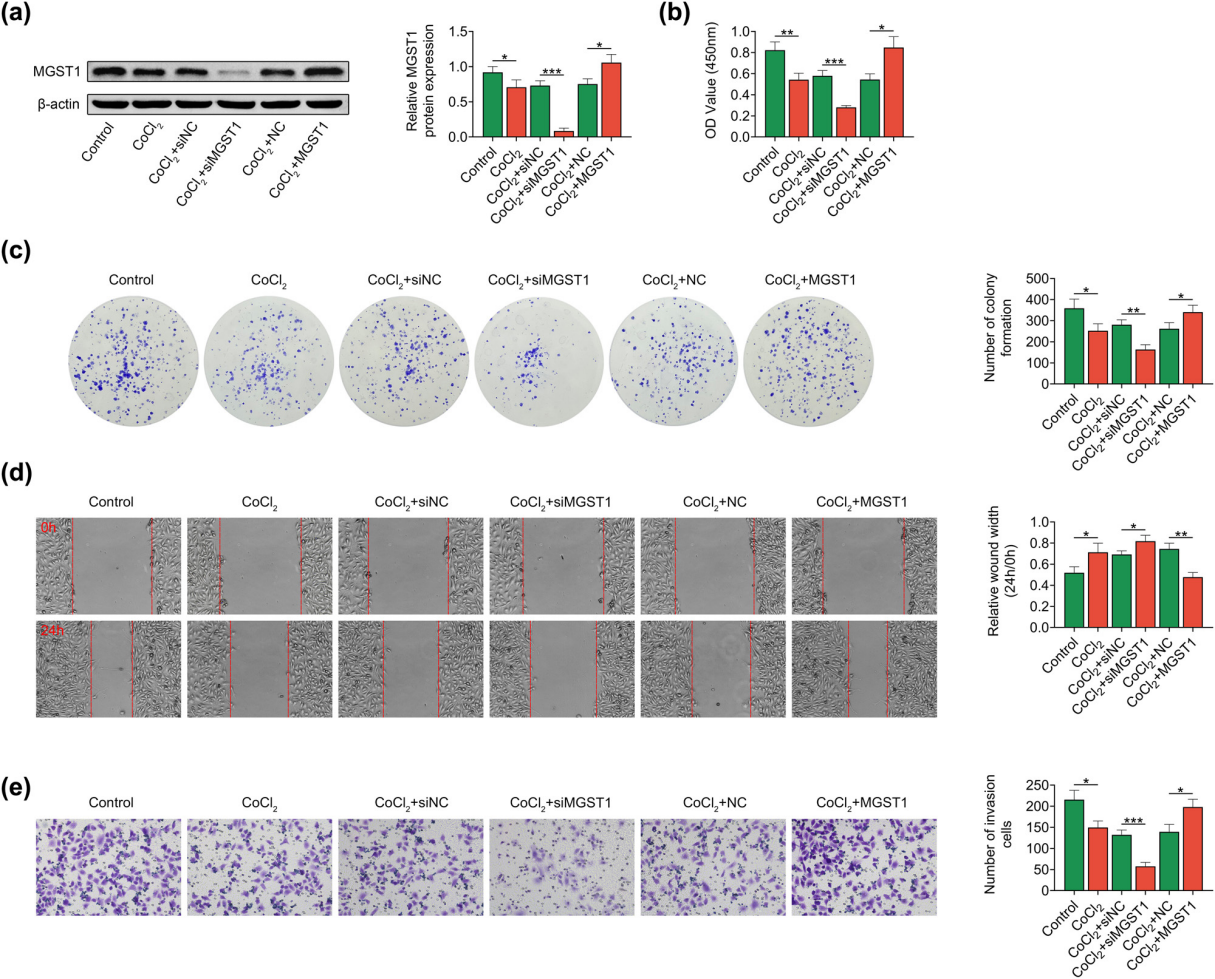
Overexpression of MGST1 increased the hypoxia-induced trophoblast cell proliferation, migration, and invasion. Groups were divided into the control, CoCl_2_, CoCl_2_ + siNC, CoCl_2_ + siMGST1, CoCl_2_ + NC, and CoCl_2_ + MGST1 group. (a) The protein level of MGST1 was tested via Western blot analysis. (b and c) The proliferation of HTR8/SVneo cells was assessed via the CCK-8 and colony formation assays. (d and e) The wound healing and Transwell assay unveiled that the elevation of MGST1 led to the increase of trophoblast cell migration and invasion. ^*^ *P* < 0.05, ^**^ *P* < 0.01, ^***^ *P* < 0.001.

Next, the function of MGST1 in PE was investigated. The cell model was established by simulating hypoxia in trophoblast cells using 250 μM CoCl_2_ for 24 h. The results indicated that MGST1 expression was downregulated by the treatment with CoCl_2_. siMGST1 transfection markedly decreased while MGST1 overexpression obviously increased the MGST1 protein level in CoCl_2_-treated trophoblast cells (Figure 2a). The results from CCK-8 assay showed that the treatment with CoCl_2_ suppressed the viability of trophoblast cells, and downregulation of MGST1 inhibited whereas upregulation of MGST 1 elevated the viability of CoCl_2_-treated trophoblast cells (Figure 2b). The colony formation assay also unveiled that the proliferation of trophoblast cells was inhibited by CoCl_2_ induction, and the proliferation of trophoblast cells was alleviated by downregulation of MGST1 and enhanced by overexpression of MGST1 in CoCl_2_-treated trophoblast cells (Figure 2c). Moreover, the wound closure was inhibited by CoCl_2_ induction, and in CoCl_2_-treated trophoblast cells, the wound closure was repressed by MGST1 knockdown and enhanced by overexpression of MGST1 (Figure 2d). The invasion of trophoblast cells was relieved by the induction of CoCl_2_, and the invasion of CoCl_2_-treated trophoblast cells was attenuated by MGST1 inhibition and promoted by MGST1 upregulation (Figure 2e). Taken together, overexpression of MGST1 increased hypoxia-induced trophoblast cell proliferation, migration, and invasion.

### 3.3 MGST1 upregulation alleviated the hypoxia-induced trophoblast cell oxidative stress

**Figure 3:**
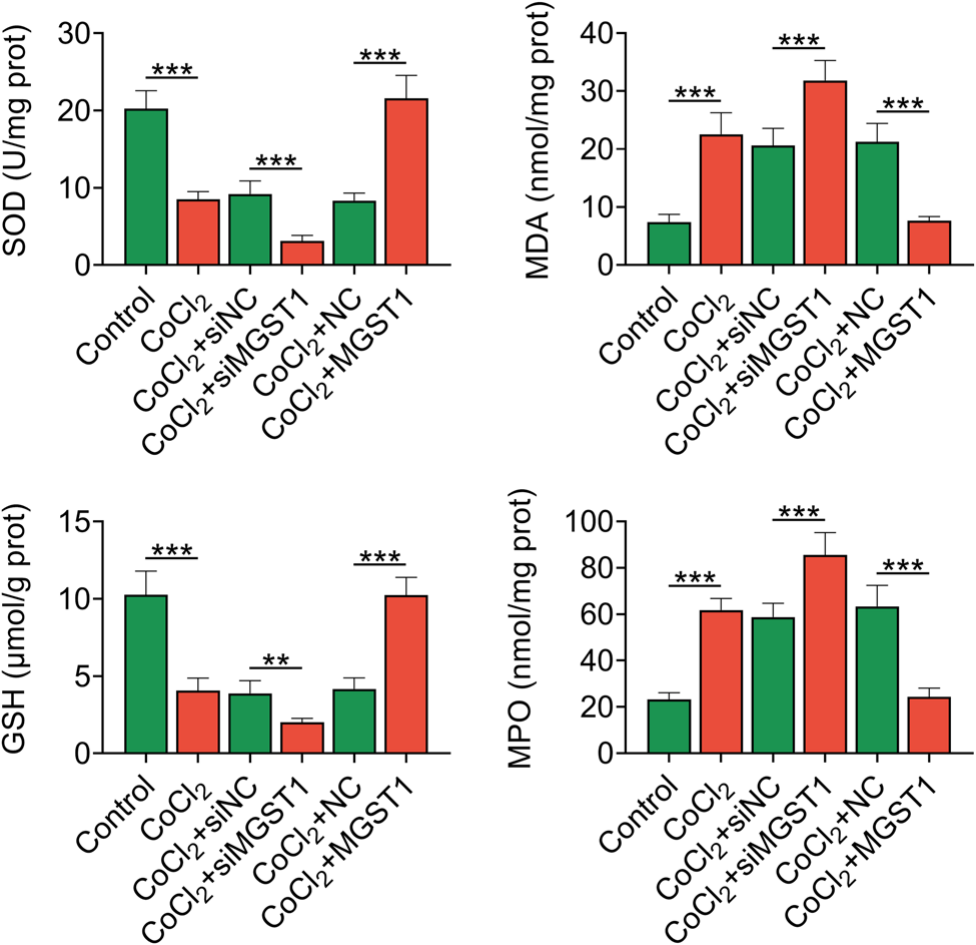
MGST1 upregulation alleviated the hypoxia-induced oxidative stress in trophoblast cell. Groups were divided into the control, CoCl_2_, CoCl_2_ + siNC, CoCl_2_ + siMGST1, CoCl_2_ + NC, and CoCl_2_ + MGST1 group. ELISA unveiled the concentrations of oxidative stress-related proteins (SOD, MDA, GSH, and MPO). ^**^ *P* < 0.01, ^***^ *P* < 0.001.

Subsequently, whether MGST1 was involved in the hypoxia-induced trophoblast cell oxidative stress was explored. CoCl_2_ treatment decreased the concentrations of SOD and GSH but increased the concentrations of MDA and MPO. The downregulation of MGST1 suppressed the concentrations of SOD and GSH, whereas the overexpression of MGST1 had the opposite effects. The concentrations of MDA and MPO were elevated by MGST1 knockdown and were repressed by MGST1 overexpression (Figure 3). These results suggested that upregulation of MGST1 alleviated the hypoxia-induced oxidative stress in trophoblast cell.

### 3.4 MGST1 activated PI3K/AKT/mTOR pathway

**Figure 4:**
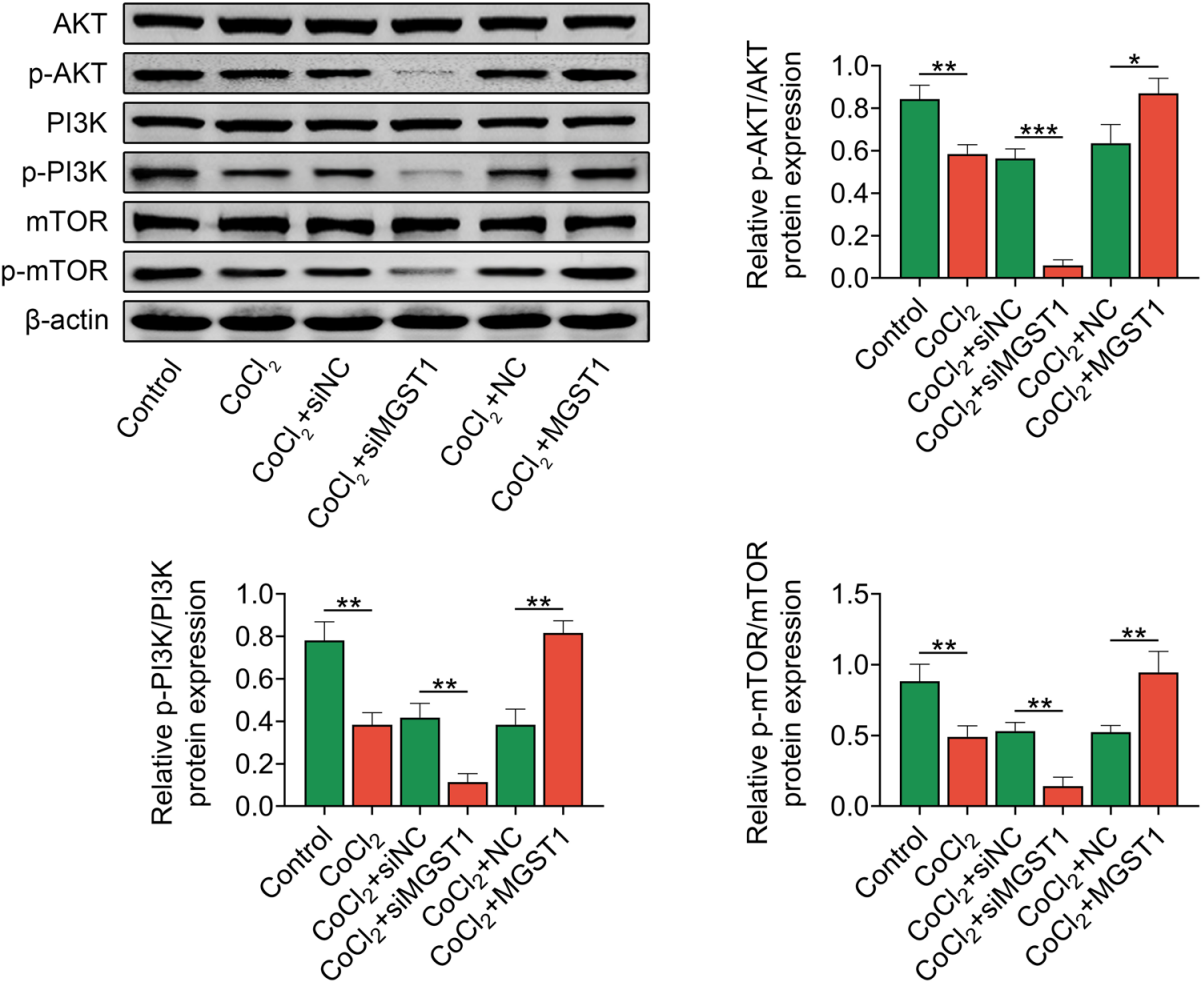
MGST1 activated PI3K/AKT/mTOR pathway. Groups were divided into the control, CoCl_2_, CoCl_2_ + siNC, CoCl_2_ + siMGST1, CoCl_2_ + NC, and CoCl_2_ + MGST1 group. Western blot analysis assessed the levels of PI3K/AKT/mTOR pathway-associated proteins (AKT, p-AKT, PI3K, P-PI3K, mTOR, and p-mTOR). ^*^ *P* < 0.05, ^**^ *P* < 0.01, ^***^ *P* < 0.001.

Then, whether MGST1 modulated PE development via the PI3K/AKT/mTOR pathway was probed. Western blot analysis uncovered that the levels of key proteins of the PI3K/AKT/mTOR pathway (p-AKT, p-PI3K, and p-mTOR) were decreased by CoCl_2_ treatment, and MGST1 downregulation decreased the protein levels of p-AKT, p-PI3K, and p-mTOR while overexpression of MGST1 enhanced their levels (Figure 4). To sum up, MGST1 activated the PI3K/AKT/mTOR pathway.

### 3.5 MGST1 modulated PE progression through the PI3K/AKT/mTOR pathway

**Figure 5:**
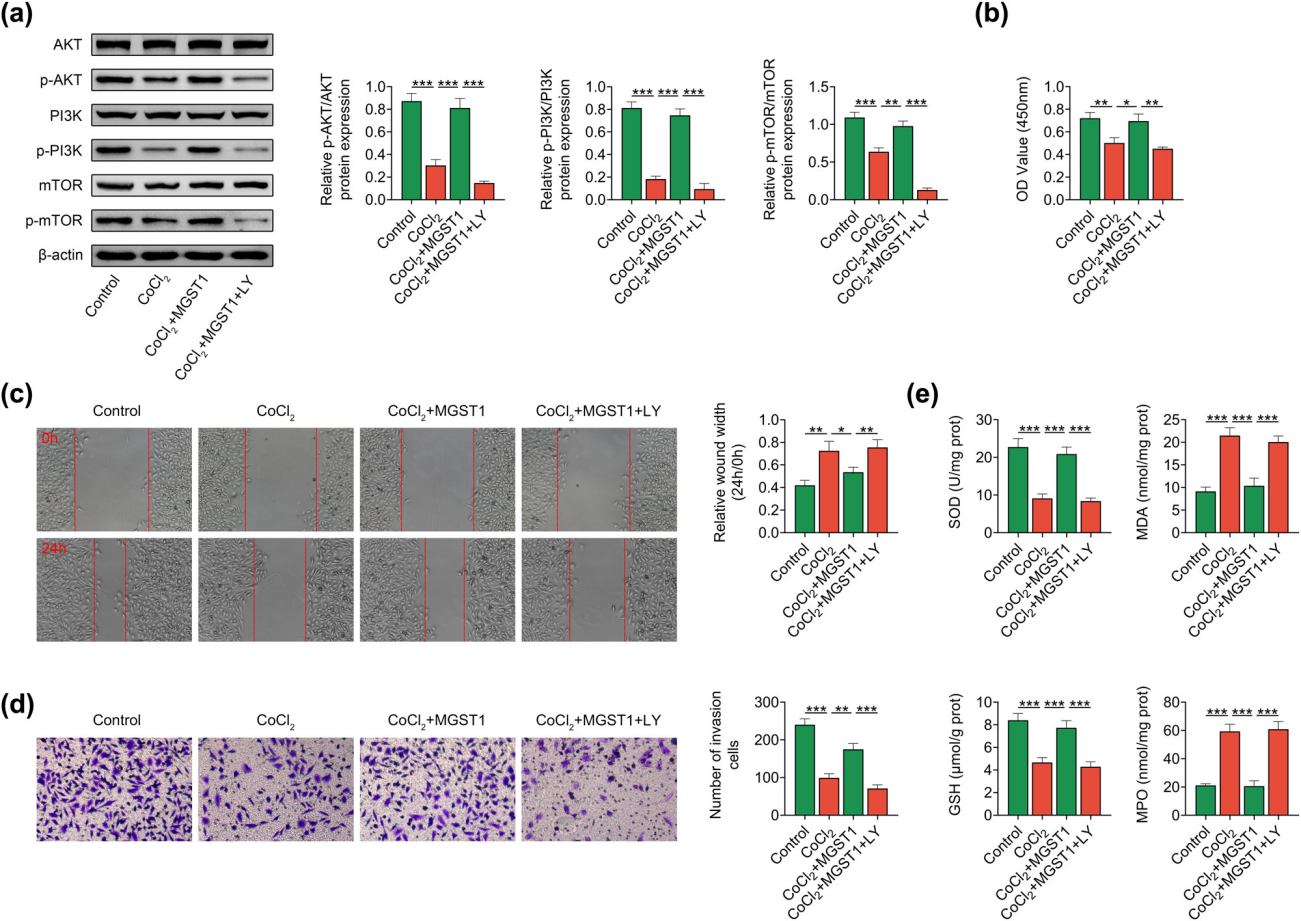
The effects of MGST1 overexpression on PE progression were attenuated after inhibiting the PI3K/AKT/mTOR pathway. Groups were divided into the control, CoCl_2_, CoCl_2_ + MGST1, and CoCl_2_ + MGST1 + LY groups. LY294002 (LY) is the inhibitor for PI3K/AKT pathway. (a) Western blot analysis detected the levels of PI3K/AKT/mTOR pathway-associated proteins (AKT, p-AKT, PI3K, P-PI3K, mTOR, and p-mTOR). (b) The cell proliferation was assessed through CCK-8 assay. (c and d) The cell migration and invasion were evaluated through wound healing and Transwell assays. (e) The concentrations of oxidative stress-related proteins (SOD, MDA, GSH, and MPO) were tested through ELISA. ^*^ *P* < 0.05, ^**^ *P* < 0.01, ^***^ *P* < 0.001.

Further experiments were conducted to verify whether MGST1 modulated PE progression through the PI3K/AKT/mTOR pathway. LY294002 (LY, the inhibitor for the PI3K/AKT pathway) was used in this work. The levels of p-AKT/AKT, p-PI3K/PI3K, and p-mTOR/mTOR were all increased after MGST1 overexpression, but these effects were attenuated by LY treatment (Figure 5a). In addition, the increased cell proliferation mediated by MGST1 overexpression was reversed by LY treatment (Figure 5b). The cell migration and invasion were enhanced by overexpression of MGST1, but these changes were attenuated by LY treatment (Figure 5c and d). Finally, the increased SOD and GSH levels, as well as the decreased MDA and MPO levels induced by MGST1 overexpression, were reversed by LY treatment (Figure 5e). These results indicated that MGST1 modulated PE progression through the PI3K/AKT/mTOR pathway.

## 4 Discussion

In this study, HTR8/SVneo cells were incubated with CoCl_2_ (250 µM) to mimic hypoxia in trophoblasts. RT-qPCR revealed that MGST1 was dramatically reduced in the placenta of PE patients. The proliferation of HTR8/SVneo cells was assessed via the CCK-8 and colony formation assays, and the results uncovered that the upregulation of MGST1 increased the cell viability of HTR8/SVneo cells. In addition, wound healing and Transwell assays unveiled that the elevation of MGST1 enhanced trophoblast cell migration and invasion. Moreover, the upregulation of MGST1 alleviated the hypoxia-induced oxidative stress in trophoblast cell. Mechanically, it was found that MGST1 modulated PE progression by activating the PI3K/AKT/mTOR pathway.

As a hypertensive disorder during pregnancy, PE is already confirmed to have various acute and long-term complications in pregnant women and newborns [16]. PE, characterized by hypertension and proteinuria, often occurs after 20 weeks of gestation, which is possibly due to inadequate blood perfusion and ischemia caused by defective placentation [17]. In previous studies, numerous researchers have found that some proteins are associated with PE. For instance, as a target of miR-485-5p, absent in melanoma 2 (AIM2) is involved in PE development by regulating the Treg/Th17 imbalance and/or AIM2 axis [18]. The silencing of histone deacetylase 4 promotes the autophagy and apoptosis of cells in PE by acting as a target of miR-29b [19]. Tumor necrosis factor-related apoptosis-inducing ligand is involved in the development of PE via modulating the invasion of trophoblast cells [20]. Although MGST1 was found to exert a role in reactive intermediate-induced injury [12], oxidative stress [21], aging [13], glioma [14], and lung adenocarcinoma [15], its function in PE remains to be explored. Here, our results showed that the level of MGST1 was decreased in the placental tissues of patients with PE. In addition, overexpression of MGST1 enhanced trophoblast cell proliferation, migration, and invasion. Moreover, the upregulation of MGST1 alleviated the hypoxia-induced oxidative stress in trophoblast cell. In summary, MGST1 was downregulated in the placenta of PE patients and modulated hypoxia-induced trophoblast cell proliferation, migration, and invasion, as well as oxidative stress in PE.

The PI3K/AKT/mTOR signaling pathway is widely accepted to be implicated with the process of normal cell growth, angiogenesis, autophagy, and metabolism [22,23,24,25]. Over-activation of the PI3K/AKT/mTOR pathway causes normal cellular dysregulation, which then leads to competitive growth, metabolic advantage, and angiogenesis [26]. The PI3K/AKT/mTOR pathway can function by interacting with some other pathways. Previously, there has been large-scale evidence indicating the role of the PI3K/AKT/mTOR pathway in various diseases. For instance, the PI3K/AKT/mTOR pathway is mediated by reactive oxygen species and exhibits a role in salidroside-modulated lipopolysaccharide-induced myocardial injury *in vitro* and *in vivo* [27]. The PI3K/AKT/mTOR pathway is regulated by Naringin and is involved in the development of glucocorticoid-induced osteoporosis [28]. The PI3K/AKT/mTOR pathway is implicated in AIM2-mediated proliferation, invasion, migration, and apoptosis in osteosarcoma cells [29]. This PI3K/AKT/mTOR pathway has also been investigated in PE progression. For instance, mangiferin activates the PI3K/AKT/mTOR pathway to relieve placental oxidative stress in the PE mouse model [30]. Moreover, DDX46 suppression modulates the PI3K/AKT/mTOR pathway to reduce trophoblast cell proliferation and migration in PE [31]. In the PE placenta, receptor tyrosine kinase-like orphan receptor 1 knockdown regulates the PI3K/AKT/mTOR pathway to suppress trophoblast cell proliferation, migration, and invasion [32]. In addition, the knockdown of apelin receptor early endogenous ligand modulates the PI3K/AKT/mTOR pathway to alleviate trophoblast invasion in early-onset PE [33]. Nevertheless, whether MGST1 modulated the PI3K/AKT/mTOR pathway in PE was largely unknown. Herein, downregulation of MGST1 decreased the protein levels of p-AKT, p-PI3K, and p-mTOR while overexpression of MGST1 elevated their levels. Collectively, MGST1 activated the PI3K/AKT/mTOR pathway in PE.

In conclusion, we found that MGST1 alleviated the oxidative stress of trophoblast cells induced by hypoxia/reoxygenation and promoted cell proliferation, migration, and invasion through the activation of the PI3K/AKT/mTOR pathway in PE. The findings might highlight the role of MGST1 in the prevention and treatment of PE in the future.
